# Evaluation of the In Vitro and In Vivo Efficacy of Ruthenium Polypyridyl Compounds against Breast Cancer

**DOI:** 10.3390/ijms22168916

**Published:** 2021-08-18

**Authors:** Oscar A. Lenis-Rojas, Catarina Roma-Rodrigues, Alexandra R. Fernandes, Andreia Carvalho, Sandra Cordeiro, Jorge Guerra-Varela, Laura Sánchez, Digna Vázquez-García, Margarita López-Torres, Alberto Fernández, Jesús J. Fernández

**Affiliations:** 1 Instituto de Tecnologia Química e Biológica António Xavier, ITQB, Av. da República, EAN, 2780-157 Oeiras, Portugal; oscar.rojas@itqb.unl.pt; 2 UCIBIO, Departamento Ciências da Vida, NOVA School of Science and Technology, Universidade Nova de Lisboa, Campus Caparica, 2829-516 Caparica, Portugal; catromar@fct.unl.pt (C.R.-R.); andreiacgmc93@gmail.com (A.C.); si.cordeiro@campus.fct.unl.pt (S.C.); 3 Associate Laboratory i4HB—Institute for Health and Bioeconomy, NOVA School of Science and Technology, NOVA University Lisbon, 2819-516 Caparica, Portugal; 4 Departamento de Zoología, Genética y Antropología Física. Facultad de Veterinaria, Universidade de Santiago de Compostela, 27002 Lugo, Spain; jorge.guerra@usc.es (J.G.-V.); lauraelena.sanchez@usc.es (L.S.); 5 Preclinical Animal Models Group, Health Research Institute of Santiago de Compostela (IDIS), 15706 Santiago de Compostela, Spain; 6 Departamento de Química, Centro de Investigaciones Científicas Avanzadas (CICA), Universidade da Coruña, 15008 A Coruña, Spain; dvazquezg@udc.es (D.V.-G.); qimarga@udc.es (M.L.-T.); qiluaafl@udc.es (A.F.)

**Keywords:** ruthenium, polypyridyl compounds, cytotoxicity, cell death, cell cycle, in vivo toxicity, MCF7 zebrafish xenograft

## Abstract

The clinical success of cisplatin, carboplatin, and oxaliplatin has sparked the interest of medicinal inorganic chemistry to synthesize and study compounds with non-platinum metal centers. Despite Ru(II)–polypyridyl complexes being widely studied and well established for their antitumor properties, there are not enough in vivo studies to establish the potentiality of this type of compound. Therefore, we report to the best of our knowledge the first in vivo study of Ru(II)–polypyridyl complexes against breast cancer with promising results. In order to conduct our study, we used MCF7 zebrafish xenografts and ruthenium complexes [Ru(bipy)_2_(C_12_H_8_N_6_-N,N)][CF_3_SO_3_]_2_ **Ru1** and [{Ru(bipy)_2_}_2_(μ-C_12_H_8_N_6_-N,N)][CF_3_SO_3_]_4_ **Ru2**, which were recently developed by our group. **Ru1** and **Ru2** reduced the tumor size by an average of 30% without causing significant signs of lethality when administered at low doses of 1.25 mg·L^−1^. Moreover, the in vitro selectivity results were confirmed in vivo against MCF7 breast cancer cells. Surprisingly, this work suggests that both the mono- and the dinuclear Ru(II)–polypyridyl compounds have in vivo potential against breast cancer, since there were no significant differences between both treatments, highlighting **Ru1** and **Ru2** as promising chemotherapy agents in breast cancer therapy.

## 1. Introduction

Currently, cancer is a pandemic with more than 18,000,000 diagnosed cases [1], being considered the second leading cause of death worldwide. In fact, in 2020, female breast cancer surpassed lung cancer as the most diagnosed cancer with 2.3 million new cases [2], and specific types of breast cancer are today still incurable [3]. Despite the improved knowledge of breast cancer biology and the great advances in targeted and immuno-oncological therapies, cytotoxic chemotherapy, such as cisplatin and its derivatives, remains central to breast cancer treatment [3,4]. However, the high toxicity and the acquired or intrinsic drug resistance remain the main limitations in the clinical application of platinum-based treatments [5]. Thus, the development of new chemotherapeutic agents is critical for further progress in cancer treatment. In this context, ruthenium-based compounds are an attractive alternative to platinum compounds since they exhibit excellent results as potential anticancer drugs [6].

**Figure 1:**
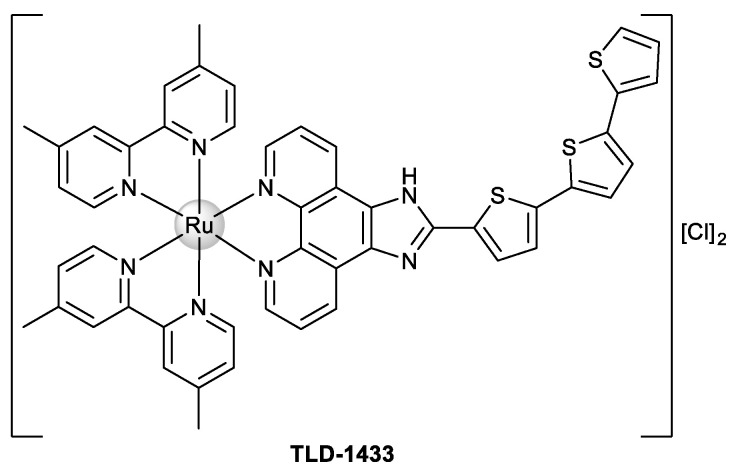
Chemical structure of Ru(II)–polypyridyl complex TLD-1433.

In particular, both mono- and dinuclear Ru(II)–polypyridyl complexes have attracted attention for the development of new therapeutic agents against cancer, and this fact is very interesting since the compound TLD-1433 (Figure 1) is in clinical trials for the treatment of noninvasive bladder cancer of the muscles (clinical trial NCT03945162) [7].

However, there are currently insufficient in vivo studies to allow for an effective evaluation of the full potential of this class of compounds [8,9]. Furthermore, for some cancers, such as breast cancer, the Ru(II)–polypyridyl complexes have been less explored [8,10]. The ruthenium compounds reported against breast cancer are generally organometallic compounds [10], e.g., ruthenium arene [11,12,13,14,15,16,17,18,19,20,21] and cyclopentadienyl [22,23,24,25,26] compounds, which have also been less explored in vivo.

Mouse xenografts are a powerful tool for drug development, but zebrafish has also attracted attention as a model in drug discovery. In particular, the larval zebrafish xenograft is ideal for drug discovery and differs fundamentally from mouse xenografts in that larval xenograft assays can be done in a short time (up to 5 days post transplant) [27,28,29,30]. There are also significant experimental advantages, including high-resolution intravital imaging and high-throughput 96-well format drug screening [27]. Moreover, there is no need for immunosuppression because larval zebrafish do not develop a functional adaptive immune system until 4–6 weeks post fertilization [29].

**Figure 2:**
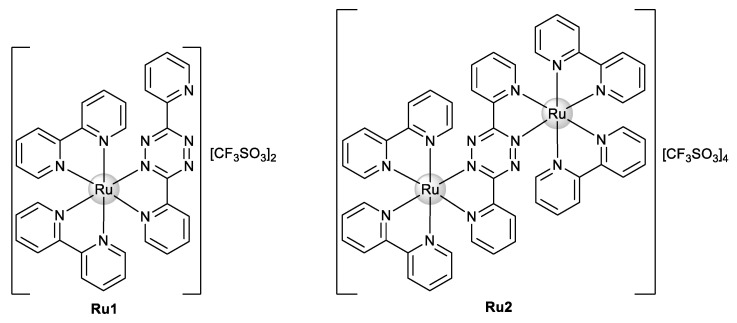
Chemical structure of Ru(II)–polypyridyl complexes **Ru1** and **Ru2**.

Our group recently synthesized and characterized mononuclear and dinuclear compounds derived from the Ru(bipy)_2_ fragment [31,32]. We demonstrated the cytotoxic activity of **Ru1** and **Ru2** (Figure 2) against human breast tumor MCF7 cells with IC_50_ values of 25.4 ± 5.0 and 30.1 ± 12.5 μM, respectively. No cytotoxicity in normal human primary fibroblasts in the case of **Ru1** was observed [31], while a cytotoxicity analysis of **Ru2** for normal cells was not performed. Both compounds interact with ctDNA, with **Ru2** specifically showing a concentration-dependent double-strand cleavage of plasmidic DNA [31,32].

As a result of the in vitro results that we obtained and the urgent need for new breast cancer treatments, we decided to not only study in more detail the anticancer activity of the Ru(II)–polypyridyl complexes **Ru1** and **Ru2** against a breast cancer cell line, MCF7, but also investigate the in vivo anticancer activity of the Ru complexes through larval zebrafish MCF7 xenografts, which to the best of our knowledge represents the first in vivo study of Ru(II)–polypyridyl complexes against breast cancer.

## 2. Results and Discussion

### 2.1. In Vitro Assays

**Figure 3:**
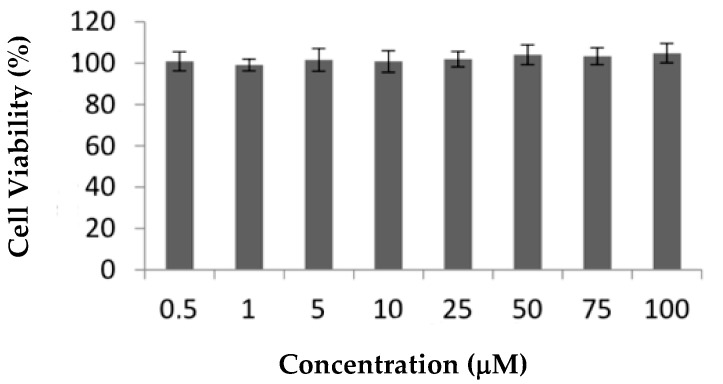
Effect of **Ru2** on cellular viability of nontumor cell line fibroblasts. Cells were exposed to 0.5–100 μM of compound or 0.1% (*v*/*v*) DMSO (vehicle control) for 72 h. The results are expressed as mean ± SEM fold-change compared to controls of at least three independent experiments.

**Ru1** and **Ru2** are promising mononuclear and dinuclear compounds, respectively, with high cytotoxicity in ovarian carcinoma cells and moderate cytotoxicity in breast adenocarcinoma MCF7 cells [31,32]. Compounds in MCF7 cells presented IC_50_ values of 25.4 ± 5.0 and 30.1 ± 12.5 μM, respectively [31,32], which are lower than the IC_50_ of cisplatin in the same cell line, 41.7 ± 1.5 μM (Supplementary Figure S1A). Here, we aimed to further explore the cellular effects and efficacy of both compounds for future breast adenocarcinoma therapy by using additional in vitro and in vivo studies. Since one of the most important drawbacks in cancer therapy is represented by the chemotherapy side-effects, we assessed the cytotoxicity of the compounds in normal human primary fibroblasts: nonepithelial human cells that synthesize the extracellular matrix and collagen, i.e., the structural framework (stroma) for animal tissues with a relevant role in the modulation of tumor microenvironment [33,34,35]. The cytotoxic effect of **Ru1** in these cells was previously described [31], while the effect of **Ru2** in terms of the cellular viability of the fibroblast cell line is presented herein for the first time (Figure 3). In contrast to cisplatin that presented an IC_50_ in fibroblasts of 8.8 ± 2.9 µM (Supplementary Figure S1B), neither Ru(II) compound displayed any in vitro cytotoxicity at concentrations up to 100 μM in human primary normal fibroblasts, reinforcing the good cytotoxic effect toward MCF7 breast adenocarcinoma cells for **Ru1** (Figure 3) [31].

### 2.2. Cell Death Mechanism

**Figure 4:**
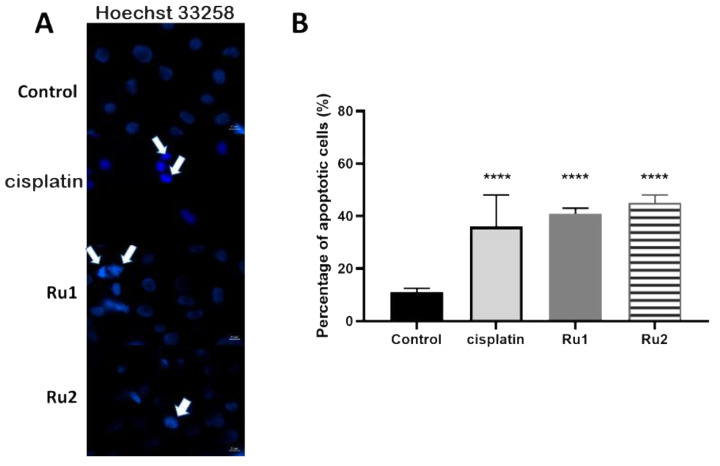
(**A**) Hoechst staining (excitation and fluorescence emission at 352 and 461 nm, respectively) of MCF7 cell line for visualization of apoptotic nuclei. Cells were grown in DMEM culture medium supplemented with 10% (*v*/*v*) fetal bovine serum in the presence of 0.1% (*v*/*v*) DMSO, the IC_50_ of cisplatin, the IC_50_ of **Ru1**, or the IC_50_ of **Ru2** for 72 h. The arrows indicate an initial event of apoptosis such as chromatin condensation and nuclear fragmentation. The images were acquired using a ZEISS Microscope with ZEN software. (**B**) Percentage of apoptotic cells in MCF7 breast adenocarcinoma cell line after exposure to 0.1% (*v*/*v*) DMSO (vehicle control) or to the IC_50_ of each Ru(II) compound. The data are presented as the mean ± SEM of three independent experiments, and the statistical significance was evaluated in relation to the reference group (control) using the one-way ANOVA method followed by a Tukey multiple comparison test (**** *p* ≤ 0.0001).

To gain insight into the mechanism of cytotoxic action induced after 72 h exposure of the MCF7 cell line to both Ru(II) mononuclear and dinuclear compounds, the level of apoptosis was evaluated by assessing Hoechst nuclei staining in the absence (DMSO as vehicle control) or presence of compounds (at IC_50_). Hoechst 33258 (2′-[4-ethoxyphenyl]-5-[4-methyl-1-piperazinyl]-2,5′-bi-1*H*-benzimidazole trihydrochloridetrihydrate) has a high affinity for nucleic acids, allowing the detection of nuclear alterations [36]. The nuclei of viable cells exhibit a blue fluorescence distributed homogeneously, whereas apoptotic cells exhibit apoptotic bodies, nuclear fragmentation, and chromatin condensation, revealing a higher fluorescence intensity [36]. As observed in Figure 4A, an increase in the number of apoptotic markers, such as fragmentation and chromatin condensation was observed in MCF7 cells incubated in the presence of both compounds, corresponding to 41% ± 3% apoptotic cells for **Ru1** (4.1-fold increase over the control) and 45% ± 4% apoptotic cells for **Ru2** (4.5-fold increase over the control) (Figure 4B).

Both compounds were previously described to induce apoptosis in ovarian carcinoma cell line A2780 [31,32]. The results presented in this study confirm that both mononuclear and dinuclear Ru(II) compounds can induce apoptosis in MCF7 cancer cells.

### 2.3. Cell-Cycle Progression

In addition to the cytotoxic effect, several antitumor drugs also demonstrate a high cytostatic potential [37,38]. To analyze the cytostatic potential of **Ru1** and **Ru2**, MCF7 cells were synchronized at the G1/S phase, and then the cell-cycle progression of untreated and compound-treated MCF7 cells (for 6 h and 12 h) was evaluated by flow cytometry using propidium iodide (PI) fluorescence.

**Figure 5:**
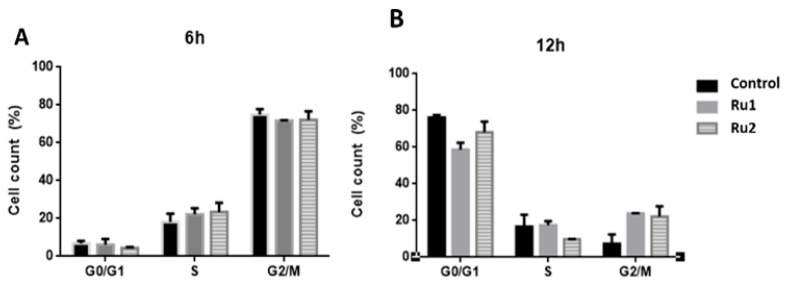
Effect of **Ru1**, **Ru2** or 0.1% (*v*/*v*) DMSO on the cell-cycle progression of MCF7 cell line. Cells were synchronized and exposed to a 0.1% (*v*/*v*) DMSO solution (vehicle control) or the IC_50_ concentrations of each compound for 6 h (**A**) and 12 h (**B**). The fluorescence content was analyzed by flow cytometry, after staining with propidium iodide (PI, excitation and fluorescence emission at 493 and 636 nm, respectively). The data are presented as the mean ± SEM of three independent experiments.

Flow cytometry analysis at 12 h showed that in untreated samples, most cells were in the G0/G1 phase (76.2%), whereas **Ru1**-treated cells showed a lower percentage of cells in the G0/G1 phase (58.7%) and an increase in the number of cells in the G2/M phase (23.9%) (Figure 5).

In the case of **Ru2**-treated cells, there was a difference in the S phase (9.8%) at 12 h when compared to control cells (16.6%), which was associated with an increase in the number of cells in the G2/M phase (22.1%) (Figure 5).

These results suggest that both compounds cause a slight delay in MCF7 cell-cycle progression, which agrees with our previous results, where both **Ru1** and **Ru2** showed the ability to interact in vitro with DNA [31,32].

### 2.4. Subcellular Distribution

**Figure 6:**
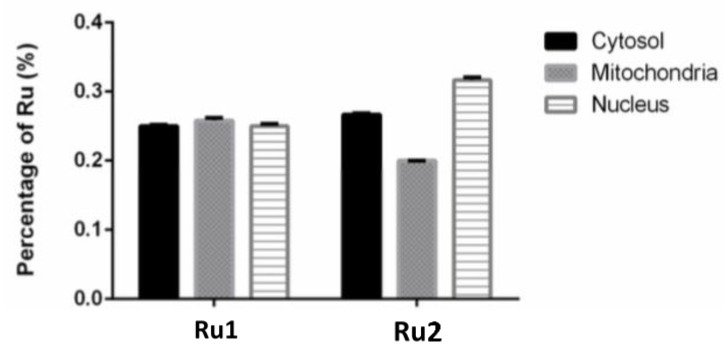
Subcellular distribution of **Ru1** and **Ru2** in MCF7 cell line. Cells were incubated in the presence of 60 μM of the complexes for 6 h. Data are presented as the mean ± SEM of two independent assays.

The knowledge of the subcellular distribution of the compound is fundamental in the elucidation of its mechanism of action [39]. The intracellular distribution of the compounds in the MCF7 cell line was studied using the Cell Fractionation Kit-Standard (ab109719, Abcam, Cambridge, UK). This kit allows the separation of cytosolic, mitochondrial, and nuclear fractions using detergents that take advantage of the characteristics and composition of different cell membranes. For both samples, the ruthenium content was determined using inductively coupled plasma atomic emission spectroscopy (ICP-AES) technique. We used at least 2× the IC_50_ concentration since the detection limits of ICP-AES did not allow us to use their respective IC_50_. After 6 h incubation, only 0.76% of **Ru1** and 0.71% of **Ru2** was found to be internalized by cells. As observed in Figure 6, when only considering the percentage of internalized compounds, both **Ru1** and **Ru2** were distributed among the cytosolic, mitochondrial, and nuclear fractions. No significant difference was observed in the amount of ruthenium among the three subcellular fractions (Figure 6). This distribution among the different fractions might indicate that these compounds might target different cellular components, in addition to DNA, which may contribute to the mechanism underlying their antiproliferative effect.

### 2.5. In Vivo Assays

**Figure 7:**
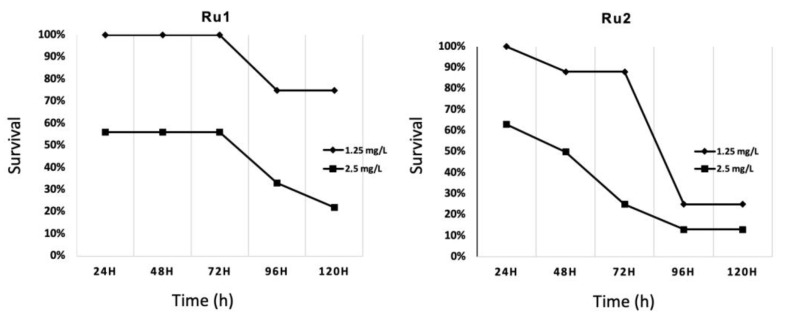
Survival evolution through time of 24 h post fertilization (hpf) zebrafish embryos exposed to ruthenium compounds at different concentrations; higher concentrations (5.0 and 10.0 mg·L^−1^) showed no survival at 24 h post treatment (hpt).

Preliminary toxicity assays on 48 h post fertilization (hpf) embryos at 34 °C were carried out in this work with the LC_10_ (lethal dose 10%) obtained from previous analyses [30,31]. This led to very high mortality rates. Then, new conditions were evaluated to identify the highest safe concentration in zebrafish embryos from 48 hpf onward, at 34 °C, dissolved in 1% (*v*/*v*) DMSO/water. In the end, the same concentration was chosen for both compounds (1.25 mg·L^−1^), showing low mortality rates for the first 72 h of exposition (Figure 7). No significant effects were found compared to controls for this period of time. For longer exposures, mortality was higher in those embryos exposed to any concentration of the drugs than the control.

**Figure 8:**
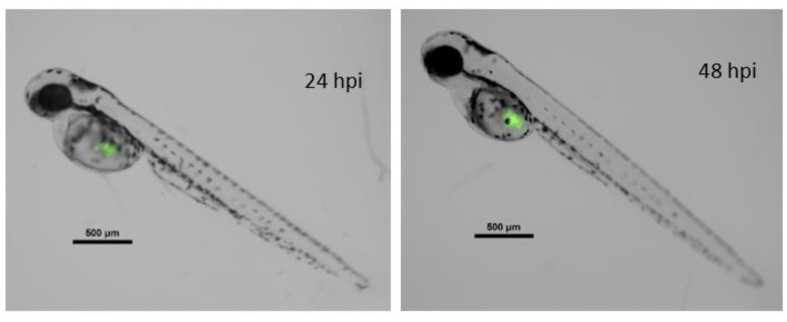
Merged composition (black/white and green fluorescence channels) of a zebrafish embryo xenografted with fluorescent MCF7 cancer line. Left image, 72 hpf zebrafish at 24 hpi. Right image, the same embryo 24 h later. hpi = hours post injection; hpf = hours post fertilization. Scale = 500 µm.

Xenografted zebrafish embryos were separated into three different treatments. One served as the control, with no drug exposition, whereas the two remaining groups were exposed to 1.25 mg·L^−1^ concentrations of **Ru1** or **Ru2**. As expected, survival in fish groups did not show differences from controls. Cell proliferation was determined by the ratio of cancer cells (i.e., fluorescence) at 24 h post antitumor treatment to that 0 h post treatment (hpt) (Figure 8).

**Figure 9:**
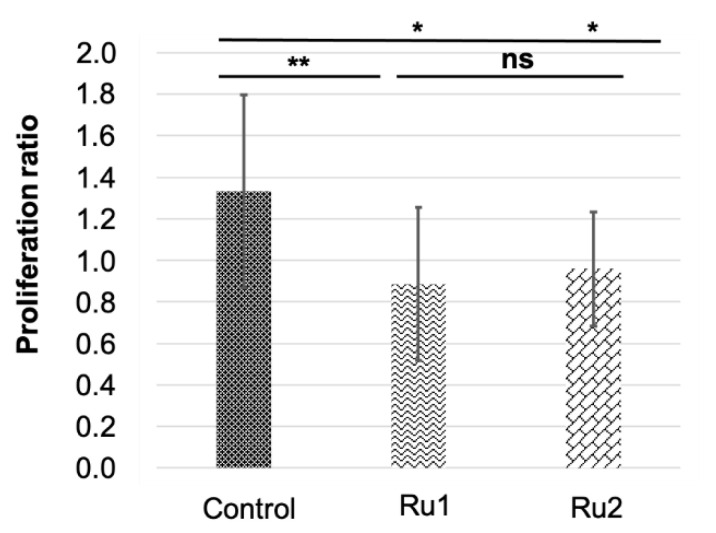
Comparison of MCF7 tumor cell variation in zebrafish embryos in control individuals and those exposed to antitumor drugs. * *p* < 0.05; ** *p* < 0.01; ns = not significant. Bars indicate standard deviation.

Then, an outlier analysis was run, which detected one outlier in the control group, none in the **Ru1** group, and three in the **Ru2** group. Results indicated that both experimental treatments significantly reduced the cancer cell population in the embryos (0.89 ± 0.37 and 0.96 ± 0.27 for **Ru1** and **Ru2** respectively) compared to controls (1.33 ± 0.46). No statistical differences were found between treatments, showing a slightly bigger reduction for **Ru1** (Figure 9).

Therefore, from the model species point of view, xenograft assays demonstrated that both newly developed antitumor drugs effectively prevented MFC7 proliferation, supporting the medicinal properties recognized for ruthenium–polypyridyl complex-based compounds [40,41,42]. Lastly, given the genetic and molecular similarities between zebrafish and human beings [43], and the predictability of zebrafish for the effect of drugs on other species, including humans [44], the compounds studied prove to be potential candidates for chemotherapeutic agents because of their safety and effectiveness. Therefore, both compounds demonstrated potential for further in vivo mice biological studies, as well as for their use as lead scaffolds for further chemical modifications to improve drug-like properties.

## 3. Materials and Methods

### 3.1. In Vitro Assays

#### 3.1.1. Cell Culture and Maintenance

The MCF7 cell line (breast cancer) expressing green fluorescent protein (GFP) (Cell Biolabs, Inc, San Diego, CA, USA) was used in this work. MCF7 cancer cells were cultured in Dulbecco’s modified Eagle’s medium (DMEM) (Invitrogen, NY, USA) supplemented with 10% (*v*/*v*) fetal bovine serum (FBS) and 1% (*v*/*v*) penicillin–streptomycin (Invitrogen, New York, NY, USA) and maintained at 37 °C in a humidified atmosphere of 5% (*v*/*v*) CO_2_. Normal human primary fibroblasts were grown in the same conditions as the MCF7 cell line, supplemented with 1% (*v*/*v*) MEM nonessential amino acids (Invitrogen) [35,45]. The fibroblast cell line was purchased from ATCC (www.atcc.org, 28 June 2021).

#### 3.1.2. Cytotoxic Activity Assay in Normal Human Fibroblasts and MCF7

The antiproliferative activity was evaluated in human normal fibroblasts using the CellTiter 96^®^ Aqueous Non-Radioactive Cell Proliferation Assay (Promega, Madison, WI, USA) and the 3-(4,5-dimethylthiazol-2-yl)-5-(3-carboxymethoxyphenyl)-2-(4-sulfophenyl)-2*H*-tetrazolium inner salt (MTS) as previously described [35,45]. MCF7 cells and normal human fibroblasts were plated into 96-well plates at 0.75 × 10^5^ cells/well and allowed to adhere for 24 h. Then, DMEM medium was removed and replaced with fresh medium containing 0.5–100 μM compounds, 0.1–100 μM cisplatin, 0.1% (*v*/*v*) DMSO (vehicle control of compounds), or 0.09% (*w*/*v*) NaCl (vehicle control of cisplatin) and incubated in the same conditions. After 72 h of treatment, the medium was replaced by the MTS solution and the subsequent experimental procedures followed a previously described method [37]. Cell viability was evaluated by measuring the absorbance at 490 nm using a Bio-Rad microplate reader, model 680 (Bio-Rad, Hercules, CA, USA).

#### 3.1.3. Hoechst 33258 Staining

MCF7 cells were collected and plated in a 24-well culture slide at 0.75 × 10^5^ cells/mL. The culture medium was removed 24 h after plating and replaced with the IC_50_ of cisplatin (positive control), **Ru1,** or **Ru2,** or 0.1% (*v*/*v*) DMSO (vehicle control) diluted in fresh medium. Following 72 h of treatment, cells were stained with Hoechst 33258 (excitation and fluorescence emission at 352 and 461 nm, respectively) in the absence of light for 15 min, at room temperature, according to the procedure described in [46]. Fluorescent nuclei were analyzed on the basis of the chromatin condensation degree and other characteristics. Normal nuclei showed uncondensed chromatin uniformly distributed over the entire nucleus. Apoptotic nuclei showed condensed or fragmented chromatin. In addition, some cells formed apoptotic bodies. The samples were photographed using a Zeiss fluorescence microscope and the photographs were acquired with Zen Blue edition software. Three random microscopic fields per sample with ca. 50 nuclei were counted. Mean values were expressed as the percentage of apoptotic nuclei.

#### 3.1.4. Cell-Cycle Analysis

MCF7 cells were seeded into an eight-well culture slide at 1 × 10^5^ cells/mL and incubated for 24 h at 37 °C, 99% (*v*/*v*) humidity, and 5% (*v*/*v*) CO_2_, before being synchronized in the early S phase by double thymidine block (2 mM) (Sigma, St. Louis, MO, USA) as described previously [45]. Cells were released from the second block by substituting with fresh medium containing the IC_50_ concentration of **Ru1** or **Ru2**, or 0.1% (*v*/*v*) DMSO (vehicle control), and they were incubated for 6 or 12 h at 37 °C and 5% (*v*/*v*) CO_2_. For synchronization control purposes, cells from another disc were collected after thymidine block. After each time point, cells were trypsinized with TrypLE^TM^ Express and centrifuged (5 min, 650× *g*, 4 °C). Supernatants were removed, and cell pellets were resuspended in phosphate-buffered saline (PBS) 1×. An additional centrifugation was performed (5 min, 650× *g*, 4 °C), and the pellets were resuspended in PBS 1× and ethanol 80% (*v*/*v*) (1:10 proportion). The ethanol solution was added gently with constant agitation. Cells were stored at 4 °C for at least 12 h. After incubation, cells were centrifuged (10 min, 5000× *g*, 4 °C), and the pellet was treated with 50 μg/mL RNase A for 30 min at 37 °C and PI (25 μg/mL, excitation and fluorescence emission at 493 and 636 nm, respectively). The DNA content was analyzed using an Attune^®^ Acoustic Focusing Flow Cytometer (Applied Biosystems, Waltham, MA, USA), and the data collected were analyzed with FCS Express 6 Flow Cytometry software.

#### 3.1.5. Subcellular Distribution

For subcellular fractionation, MCF7 cells were seeded in 25 cm^2^ culture flasks at a cell density of 1 × 10^6^ cells/flask and incubated for 24 h at 37 °C, 99% (*v*/*v*) humidity, and 5% (*v*/*v*) CO_2_. After 24 h incubation, the medium was replaced with 5 mL of fresh medium containing 60 μM **Ru1** or **Ru2** (a concentration high enough to be detected) and incubated for 6 h at 37 °C. After incubation, the supernatant was collected; cells were detached from the culture flask and pelleted at 500× *g* for 5 min. Then, the cytosolic, mitochondrial and nuclear fractions were sequentially isolated using the Cell Fractionation Kit Standard (Abcam, Cambridge, MA, USA) according to the manufacturer’s instructions. Afterward, aqua-regia (HNO₃ and HCl in a 1:3 proportion) was added to the four fractions in 1:2 proportion (aqua regia–sample volume) and samples were incubated overnight at room temperature. The four fractions were then analyzed using a Horiba Jobin Yvon inductively coupled plasma atomic emission spectrometer as a paid service (Analytical Laboratory, Department of Chemistry, FCT-UNL) to determine the amount of ruthenium present in each sample.

#### 3.1.6. Statistical Analysis

All data were expressed as the mean ± SEM of at least three independent biological experiments. Statistical significance was evaluated using Student’s *t*-test and one-way ANOVA followed by a Tukey multiple comparison test; a *p*-value <0.05 was considered statistically significant.

### 3.2. In Vivo Assays

#### 3.2.1. Zebrafish Maintenance

Adult zebrafish (wild-type) were maintained at 28.5 °C with a light/dark cycle of 14/10 h following The Zebrafish Book recommendations (Westerfield, 2007). Embryos were obtained as massive spawning and were maintained in purified and dechlorinated water (water). In both toxicity and xenograft experiments, zebrafish embryos were kept at 28 °C until the experiments began, at which point the embryos were raised at 34 °C.

#### 3.2.2. Toxicological Analysis

Toxicological analyses were previously developed on zebrafish embryos for full characterization of the compounds [30,31], following the OECD standardized protocol [47]. Nevertheless, due to the differences in the experimental conditions applied in the original toxicological evaluations to those applied here, new assays were developed. The embryo initial stage was delayed from 0 hpf to 48 hpf, the temperature was raised from 28 to 34 °C in 96-well plates, and the compounds were dissolved in 1% (*v*/*v*) DMSO water. After preliminary trials, both compounds were tested at 1.25, 2.50, 5, and 10 mg·L^−1^. Survival was checked every 24 h from 0 hpt (i.e., 48 hpf) to 120 hpt (168 hpf). The highest concentration without significant effects (high phenotypic effects or low survival) was chosen for each compound to expose the xenografted embryos and to evaluate the effect on the cancer cell population.

#### 3.2.3. Zebrafish Xenografts

The MCF7 cell line expressing GFP was used in this work to develop the xenograft model. Breast cancer cells were cultured in DMEM supplemented with 10% (*v*/*v*) FBS and 1% (*w*/*v*) penicillin–streptomycin (Gibco, ThermoFisher Scientific, Waltham, MA, USA). Cells were detached from the culture flask, and approximately 1 × 10^6^ cells were concentrated in 10 μL of 2% PVP40 (Sigma, Madrid, Spain) in PBS. Next, 15–20 nL of this cell suspension was microinjected in the yolk of 48 hpf zebrafish embryos. To improve the proliferation of MCF7, taking into account zebrafish embryo survival, the incubation temperature was set to 34 °C.

Embryos recovered from microinjection for 24 h, which also favored tumor cell proliferation. After that time, embryos without cells properly injected were discarded. Remaining individuals were randomly separated into three different groups: 48 individuals exposed to **Ru1**, 48 exposed to **Ru2**, and 10 noninjected and nonexposed embryos used as the control. Proper concentration of the ruthenium compounds was dissolved as in the toxicological analysis. Injected individuals were photographed just prior to drug exposition and 24 h later. Pictures were taken using a Nikon AZ100 zoom microscope, with a filter for GFP detection (509 nm). Exposition was fixed at 300 ms. Quantifish 1.0 software was used to obtain the fluorescence information of each image [47]. Then, the proliferation ratio was calculated for each individual. IBM SPSS Statistics v.24 software was used for the detection of outliers and for treatment comparisons (One-way ANOVA).

All experiments were carried out under current legislation. Zebrafish in their early stages (<120 hpf) are not protected according to the European Union Directive 2010/63/EU. Experiments were performed in agreement and with the approval of the Animal Care and Use Committee of the University of Santiago de Compostela. All surviving individuals were euthanized by tricaine (MS-222) overdose.

## 4. Conclusions

Both **Ru1** and **Ru2** complexes were active in the in vivo treatment of breast cancer. In fact, both compounds inhibited tumor growth by an average of 30% compared to control in MCF7 zebrafish xenografts. Furthermore, there were no significant signs of lethality observed. Therefore, there was in vivo selectivity toward cancer cells, and this result was also observed in vitro. Moreover, both complexes caused cell death through apoptosis pathways, along with a slight delay in cell-cycle progression. Nonetheless, it cannot be excluded that these compounds might induce other types of cell death, such as autophagy, as observed in ovarian cancer cell line A2780 after exposure to **Ru1** and **Ru2**. Subcellular distribution results suggest that DNA is not the only target for these compounds, despite their high affinity for DNA, as suggested in previously published studies [31,32]. Furthermore, both mono- and dinuclear Ru(II) compounds have potential as chemotherapy agents against breast cancer since no significant differences were found between treatments with **Ru1** and **Ru2**; consequently, this lays the foundation for the design of new ruthenium–polypyridyl compounds against cancer.

## Supplementary Materials

The following are available online at https://www.mdpi.com/article/10.3390/ijms22168916/s1.
