# Supplementary material for: Evaluation of the In Vitro and In Vivo Efficacy of Ruthenium Polypyridyl Compounds against Breast Cancer
Source: Int J Mol Sci. 2021 Aug 18;22(16):8916. doi: 10.3390/ijms22168916 (PMC8396206; doi:10.3390/ijms22168916)
Supplement: Supplementary file 1 [file ijms-22-08916-s001.zip › ijms-1313935-supplementary.pdf]

Supplementary information

Evaluation of the In Vitro and In Vivo Efficacy of Ruthenium Polypyridyl Compounds against Breast Cancer

Oscar A. Lenis-Rojas [1], Catarina Roma-Rodrigues [2,3], Alexandra R. Fernandes [2,3,*], Andreia Carvalho [2], Sandra Cordeiro [2,3], Jorge Guerra-Varela [3], Laura Sánchez [4,5], Digna Vázquez-García [6], Margarita López-Torres [6], Alberto Fernández [6], and Jesús J. Fernández [6,*]

1 Instituto de Tecnologia Química e Biológica António Xavier, ITQB, Av. da República, EAN, 2780-157 Oeiras, Portugal; oscar.rojas@itqb.unl.pt

2 UCIBIO, Departamento Ciências da Vida, NOVA School of Science and Technology, Universidade Nova de Lisboa, Campus Caparica, 2829-516 Caparica, Portugal; catromar@fct.unl.pt (C.R.-R.); andreiacgmc93@gmail.com (A.C.); si.cordeiro@campus.fct.unl.pt (S.C.)

3 Associate Laboratory i4HB—Institute for Health and Bioeconomy, NOVA School of Science and Technology, NOVA University Lisbon, 2819-516 Caparica, Portugal.

4 Departamento de Zoología, Genética y Antropología Física. Facultad de Veterinaria, Universidade de Santiago de Compostela, 27002 Lugo, Spain. jorge.guerra@usc.es (J.G.-V.); lauraelena.sanchez@usc.es (L.S.)

5 Preclinical Animal Models Group, Health Research Institute of Santiago de Compostela (IDIS) 15706 Santiago de Compostela, Spain.

6 Departamento de Química & Centro de Investigaciones Científicas Avanzadas (CICA), Universidade da Coruña, 15008 A Coruña, Spain. dvazquezg@udc.es (D.V.-G); qimarga@udc.es (M.L.-T.); qiluaafl@udc.es (A.F.)

* Correspondence: ma.fernandes@fct.unl.pt (A.R.F.); lujjfs@udc.es (J.J.F.)

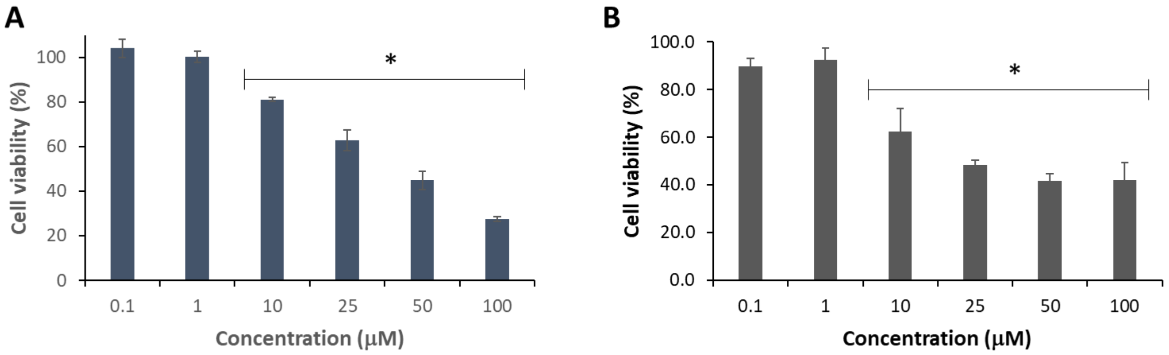

Figure S1. Effect of cisplatin on cellular viability of A) breast adenocarcinoma cell line MCF7 and B) non-tumor cell-line fibroblasts. Results are expressed as mean ± SD fold change compared to untreated cells. * p-value < 0.05.
